# Intra-articular MMP-1 in the spinal facet joint induces sustained pain and neuronal dysregulation in the DRG and spinal cord, and alters ligament kinematics under tensile loading

**DOI:** 10.3389/fbioe.2022.926675

**Published:** 2022-08-03

**Authors:** Meagan E. Ita, Sagar Singh, Harrison R. Troche, Rachel L. Welch, Beth A. Winkelstein

**Affiliations:** ^1^ Spine Pain Research Laboratory, Department of Bioengineering, University of Pennsylvania, Philadelphia, PA, United States; ^2^ Department of Neurosurgery, University of Pennsylvania, Philadelphia, PA, United States

**Keywords:** nociception, collagen, stiffness, collagenase, collagen hybridizing peptide, matrix metalloproteinase-1 (MMP-1), microstructure

## Abstract

Chronic joint pain is a major healthcare challenge with a staggering socioeconomic burden. Pain from synovial joints is mediated by the innervated collagenous capsular ligament that surrounds the joint and encodes nociceptive signals. The interstitial collagenase MMP-1 is elevated in painful joint pathologies and has many roles in collagen regulation and signal transduction. Yet, the role of MMP-1 in mediating nociception in painful joints remains poorly understood. The goal of this study was to determine whether exogenous intra-articular MMP-1 induces pain in the spinal facet joint and to investigate effects of MMP-1 on mediating the capsular ligament’s collagen network, biomechanical response, and neuronal regulation. Intra-articular MMP-1 was administered into the cervical C6/C7 facet joints of rats. Mechanical hyperalgesia quantified behavioral sensitivity before, and for 28 days after, injection. On day 28, joint tissue structure was assessed using histology. Multiscale ligament kinematics were defined under tensile loading along with microstructural changes in the collagen network. The amount of degraded collagen in ligaments was quantified and substance P expression assayed in neural tissue since it is a regulatory of nociceptive signaling. Intra-articular MMP-1 induces behavioral sensitivity that is sustained for 28 days (*p* < 0.01), absent any significant effects on the structure of joint tissues. Yet, there are changes in the ligament’s biomechanical and microstructural behavior under load. Ligaments from joints injected with MMP-1 exhibit greater displacement at yield (*p* = 0.04) and a step-like increase in the number of anomalous reorganization events of the collagen fibers during loading (*p* ≤ 0.02). Collagen hybridizing peptide, a metric of damaged collagen, is positively correlated with the spread of collagen fibers in the unloaded state after MMP-1 (*p* = 0.01) and that correlation is maintained throughout the sub-failure regime (*p* ≤ 0.03). MMP-1 injection increases substance P expression in dorsal root ganglia (*p* < 0.01) and spinal cord (*p* < 0.01) neurons. These findings suggest that MMP-1 is a likely mediator of neuronal signaling in joint pain and that MMP-1 presence in the joint space may predispose the capsular ligament to altered responses to loading. MMP-1-mediated pathways may be relevant targets for treating degenerative joint pain in cases with subtle or no evidence of structural degeneration.

## Introduction

Musculoskeletal pain is the most common type of chronic pain and cause of disability, with spine pain having the highest prevalence (Institute of Medicine, 2011). The cervical facet joints are the source in 28% of neck-pain cases (Manchikanti et al., 2015), often from trauma-induced joint degeneration and/or osteoarthritis (Gellhorn et al., 2013; Hawellek et al., 2017). Owing to its nociceptor innervation (McLain, 1994; Kallakuri et al., 2012), the ligamentous capsular ligament that encapsulates the facet acts as a pain sensor and transmits nociceptive signals under degenerative pathologic conditions (Cavanaugh et al., 2006; Manchikanti et al., 2015; Ita et al., 2017). Degeneration is caused by a complex combination of biomechanical and biological cascades that initiate nociception in innervated joint tissues like the facet capsular ligament (Gellhorn et al., 2013; Ita et al., 2017). Rodent models of joint degeneration suggest that neuropathic and inflammatory mechanisms in both the peripheral and central nervous systems play a role in degenerative joint pain (Havelin et al., 2015; Syx et al., 2018). Yet, if, and which, biologic mediators regulate the pathophysiological cascades that transmit nociceptive signals in joint-mediated pain are unknown.

Matrix metalloproteinases (MMPs) are proteases that regulate the mechanical, structural, and cellular responses of resident nerves and synovial fibroblasts in ligaments in painful joint pathology (Bartok and Firestein, 2010; Sbardella et al., 2012). The interstitial collagenase MMP-1 is a likely mediator of pathophysiological cascades in joint tissues since it is in the joint tissues after trauma (Konttinen et al., 1999; Cohen et al., 2007; Haller et al., 2015) and with degeneration (Kim et al., 2015). MMP-1 directly regulates structure and cell-signaling, but also indirectly regulates joint mechanics. For example, MMP-1 degrades extracellular matrix (ECM) components of the synovium and capsular ligament, including Type I collagen (Fields, 1991; Sbardella et al., 2012), which can alter the biomechanics of the overall joint (Otterness et al., 2000). MMP-1 also acts on neuronal receptors involved in nociception (Dumin et al., 2001; Allen et al., 2016). For example, MMP-1 forms a trimeric complex with neuronal α2β1-integrin receptors and Type I collagen that can initiate nociceptive-related cascades (Dumin et al., 2001; Allen et al., 2016). Furthermore, MMP-1 cleavage of the protease-activated receptor-1 (PAR-1) increases intracellular calcium within minutes and can thereby affect calcium-dependent neuronal signaling (Conant et al., 2002; Allen et al., 2016). Finally, MMP-1 has roles in regulatory pathways with neuropeptides and cytokines that mediate pain (Visse and Nagase, 2003; Fan et al., 2009). Despite evidence suggesting a role for MMP-1 in painful diseases, its role in joint pain is not defined. Moreover, it is unknown if MMP-1 *alone*, absent trauma or an overt degenerative state, is sufficient to induce pain.

Mechanotransduction between afferent fibers and the collagen network they are embedded in regulate nociceptive cascades following supra-physiologic loading of the capsular ligament (Zhang et al., 2016; Ban et al., 2017). For example, anomalous collagen fiber reorganization in the ligament occurs at strains that also produce behavioral sensitivity in the rat (Quinn and Winkelstein, 2007; Quinn et al., 2010), suggesting that abnormal microstructural reorganization of the capsule’s collagen network may explain pain onset with facet capsule injury. In fact, intra-articular purified bacterial collagenase in the cervical facet that induces behavioral sensitivity and neuronal dysregulation is due, at least in part, to microscale degradation of collagenous joint tissues (Ita et al., 2020). Despite the potential for injury and/or altered local mechanics from tissue trauma, the role of the collagen matrix in nociceptive signaling in degenerative pain without an inciting mechanical event, and whether MMP-1 is involved, are not clear. Further, it is unknown whether MMP-1 exposure predisposes the capsular ligament to altered biomechanical responses during loading.

We recently found increased MMP-1 in neurons in the dorsal root ganglia (DRG) and spinal cord together with increased substance P, a nociceptive neuropeptide (Pernow, 1953; Zhang et al., 2017; Zieglgänsberger, 2019), and sustained pain-like behaviors in rats 3 weeks after intra-articular bacterial collagenase in the cervical facet (Ita et al., 2020). Bacterial collagenase is not a direct substrate of, or ligand to, MMP-1. MMP-1 is regulated, in part, by neurons (Zhou et al., 2014), fibroblasts (Bartok and Firestein, 2010; Petersen et al., 2012), the ECM (Visse and Nagase, 2003), and by other MMPs (Fields, 2013). So, its increase after intra-articular bacterial collagenase (Ita et al., 2020) suggests that collagenase alters any one, or all, of these regulatory mechanisms and implicates MMP-1 in joint-mediated sensitivity. Furthermore, our prior work exploited a purified bacterial collagenase to isolate the collagenolytic activity of MMP-1 in isolation from its functions; using intra-articular MMP-1 expands on that work to include the collagenolytic and myriad non-collagenolytic roles of human MMP-1 on pain, joint structure-function, and neuronal dysregulation.

As such, this study tested whether intra-articular MMP-1 induces pain when it is introduced in the rat C6/C7 facet joint because C6/C7 is among the most common levels implicated in facet-mediated chronic pain (Panjabi et al., 1998; Ita et al., 2017). Exogenous intra-articular MMP-1 in the facet joint *alone* was hypothesized to produce behavioral sensitivity by mediating the capsular ligament’s collagen network and regulating substance P. Behavioral sensitivity was measured using mechanical hyperalgesia for 28 days after MMP-1 injection. After 28 days, histology was used to evaluate the extent of structural degradation of the joint tissues. The macroscale biomechanics, surface strain fields, and microstructural kinematics of the capsular ligament under tensile load were also tested in isolated joints after MMP-1 injection. In those tests, quantitative polarized light imaging (QPLI) was integrated to quantify microstructural changes in the collagen fiber organization and kinematics during loading (Tower et al., 2002; Quinn et al., 2010). To evaluate the effect of intra-articular MMP-1 on degradation of the capsular ligament’s ECM, a collagen hybridizing peptide (CHP) detected degraded collagen in capsular ligament tissue at day 28 after MMP-1 injection (Lin et al., 2019). Substance P was also assayed in the DRG and spinal cord at the same time.

## Materials and methods

### Animals and husbandry

All procedures were performed with University of Pennsylvania IACUC approval and under the IASP guidelines (Zimmermann, 1983). Experimental details were in accordance with the Animal Research: Reporting *In Vivo* Experiments (ARRIVE) guidelines that aim to improve standards of the reporting of animal experiments (Kilkenny et al., 2010). Studies used adult male Holtzman rats weighing 477 ± 43 g at the end of the study (HsdHot:Holtzman Sprague Dawley; Envigo; Indianapolis, IN). Male Holtzman rats were used as an established animal model for replicating clinically relevant pain-like behavior from the cervical spinal facet joints (Lee et al., 2008) and for their similarity in spinal morphology to the human (Jaumard et al., 2015).

**FIGURE 1:**
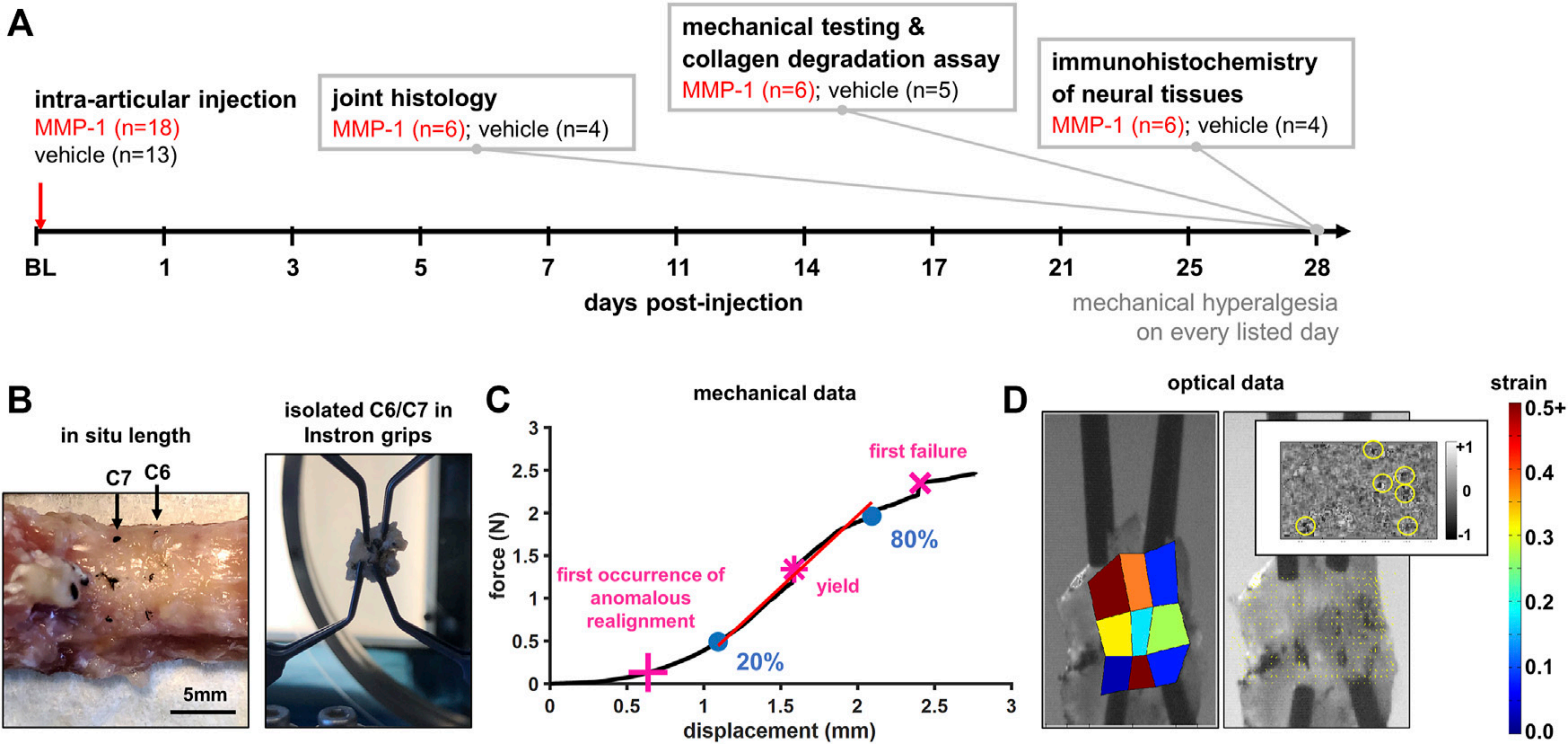
**(A)** Study design showing time course and details for measured outcomes. Rats underwent an intra-articular injection of either MMP-1 or vehicle at the start of the study (baseline; BL). Mechanical hyperalgesia was used to quantify behavioral sensitivity for 28 days. On day 28, tissue was harvested from separate groups of rats for: joint histology, mechanical testing of isolated joint tissue, assay of collagen degradation, or immunohistochemistry of neural tissues **(B)** Specimen preparation and mechanical testing. The *in situ* length across the C6/C7 motion segment of harvested spines was measured and re-established in the testing grips as the unloaded reference position for mechanical testing **(C)** Analysis of biomechanical data. An exemplar force-displacement response of a ligament from a vehicle-injected joint (Rat #54) shows relevant events: the first occurrence of anomalous realignment (+), yield (*), and first failure (x). The curve is shown to end at the point of ultimate rupture. Stiffness was calculated as a linear fit (red line) from 20% to 80% of first failure (blue circles) **(D)** Optical data analysis. An exemplar high-speed image with a corresponding maximum principal strain map, collagen fiber alignment map, and the detection of anomalous fiber reorganization events (yellow circles in inset) are shown at first failure for Rat #54.

Rats were housed in groups of two in standard polycarbonate caging (AnCare; Bellmore, NY), with 0.25-inch corncob bedding (Bed-o’Cobs; The Andersons Lab Bedding Products; Maumee, OH) and unlimited access to food (LabDiet 5001; LabDiet; St Louis, MO) and water (acidified to pH = 3). Rats were housed under a 12:12 h light:dark cycle in a temperature- and humidity-controlled environment in accordance with recommendations in The Guide for Care and Use of Laboratory Animals (National Research Council, 2011). All rats were housed in the same facility and cages were rearranged randomly throughout the duration of the study following the daily monitoring checks. A total of 34 animals were used in this study. Sample sizes for this study were based on the minimum sample size of n = 6 per group necessary to determine differences in mechanical hyperalgesia between two experimental groups (Ita et al., 2020) and the consideration of harvested tissues being designated toward three separate types of assays (Figure 1A).

### Intra-articular injections

Surgeries were performed under inhalation isoflurane anesthesia (4% induction; 2.5% maintenance) in a designated surgical suite adjacent to the animal housing facility. To expose the facet joints, a midline incision was made extending from the C4 cervical to the T2 thoracic vertebra and paraspinal musculature was cleared (Ita et al., 2020). The bilateral C6/C7 facet joints were finely dissected and injected bilaterally with either human recombinant MMP-1 (30 μg/ml; SRP3117; Sigma; St. Louis, MO) dissolved in sterile water (10μL; MMP-1 n = 18) or only sterile water (10μL; vehicle n = 13) using a 33 gauge hypodermic needle (TSK Laboratory; Japan) and 10 μL glass syringe (Kent Scientific Corporation; Torrington, CT) (Figure 1A), using reported methods (Kras et al., 2015; Ita et al., 2020). Human recombinant MMP-1 was chosen because it has been shown to activate rodent receptors (Tressel et al., 2011; Foley et al., 2013) and has analogous functions to the MMP-1a ortholog in the rat (Balbín et al., 2001). Rats were assigned to receive either an MMP-1 or vehicle injection at random immediately following behavioral testing on the day of surgery. To blind the surgeon to the experimental group, vials containing either MMP-1 or vehicle solution were aliquoted away by a second researcher and given to the surgeon for injection in unlabeled containers. Immediately after injection, wounds were sutured and stapled, and rats were recovered in room air. Weight gain and animal welfare were monitored daily, and the surgical staples were removed after 14 days. Rat group designations were revealed following tissue harvest to only certain members of the research team; co-authors who performed histology assays and imaging, biomechanical testing, and DRG scoring of substance P immunolabeling remained blinded to rat group designations.

### Mechanical hyperalgesia

Behavioral sensitivity was assessed by measuring mechanical hyperalgesia in the bilateral forepaws of each rat before surgery (baseline) and for 28 post-operative days (Figure 1A). Behavioral testing was performed in the morning hours in a room designated exclusively to behavioral testing that was free from outside noise to mitigate confounding external stimuli. In order to acclimatize rats to the behavioral testing protocol, rats underwent at least 3 days of the mechanical hyperalgesia protocol prior to the day of surgery. It was decided *a priori* that if rats exhibited hypersensitivity (exhibited by a baseline paw withdrawal threshold less than 8) then they would be excluded from the study. No rats in this study exhibited hypersensitivity during behavioral testing acclimatization. A blinded tester measured the paw withdrawal threshold (PWT) in both forepaws in response to stimulation using a series of von Frey filaments (Stoelting; Wood Dale, IL) with increasing strength (1.4–26 g) (Kras et al., 2015; Ita et al., 2020). Each filament was separately applied five times to each forepaw, and a positive response was recorded if the rat exhibited an abrupt withdrawing of its forepaw or withdrawing coupled with nocifensive behaviors of licking or shaking when stimulated. Once a positive response was recorded for two consecutive filaments, the lower strength filament was taken as the PWT for that testing session. Three rounds of testing were completed on each day, separated by at least 10 min; all rounds were averaged across rats in each group for both the left and right PWTs on each day.

### Tissue harvest

On day 28 after behavioral testing, rats were anesthetized with sodium pentobarbital (65 mg/kg; i.p.) and underwent transcardial perfusion with phosphate-buffered saline (PBS; 250 ml) followed by 4% paraformaldehyde (PFA; 250 ml) (MMP-1 n = 12; vehicle n = 8), or PBS only (n = 6 MMP-1; n = 5 vehicle) (Figure 1A). In separate groups of rats, tissue was harvested to assess joint histology, neural immunohistochemical (IHC) labeling, or biomechanical responses and CHP signal (Figure 1A). To enable histological analyses, spinal columns from the occiput to T2 (MMP-1 n = 6; vehicle n = 4) were harvested from PFA-perfused rats (Figure 1A). Fixed spinal columns were post-fixed in 4% PFA for 24 h s, held in 30% sucrose (Sigma; St. Louis, MO) in PBS for 7 days, and decalcified in 10% EDTA (Thermo Fisher; Waltham, MA) for 3 weeks (Kras et al., 2015; Ita et al., 2020). The bilateral C6/C7 facet joints were embedded in Tissue-Tek OCT Compound (Fisher Scientific; Waltham, MA), coronally cryosectioned (16 μm), and thaw-mounted onto Superfrost Plus slides (Fisher Scientific). From a separate group of fixed rats (MMP-1 n = 6; vehicle: n = 4), cervical DRG and spinal cord tissues were harvested from C5 to C8 for IHC assays on neural tissue (Figure 1A). Tissues at C7 were post-fixed in PFA for 24 h, held in 30% sucrose for 7 days, and embedded in OCT (Fisher Scientific). Axial cryosections (14μm; 6–8/rat) of DRG and spinal cord sections were thaw-mounted onto slides. Joint and neural tissue from naïve rats (n = 2) were also included to provide un-operated tissues as control samples.

Separate fresh matched spinal columns from occiput to T2 (MMP-1 n = 6; vehicle n = 5) were harvested from rats that underwent perfusion with only PBS for biomechanical testing and the CHP assay of degraded collagen (Figure 1A). Cervical spinal columns were wrapped in saline-soaked gauze and stored at −20°C; a matched spinal column from a naïve rat was also processed as a comparison for an un-operated facet joint.

### Facet joint histology

Separate C6/C7 coronal joint tissue sections were stained with Safranin O/Fast Green to visualize the joint’s cartilage and bone or Picrosirius Red/Alcian Blue to visualize collagen fibers in the ligament (Ita et al., 2020); stained sections were imaged with the 20x objective of an EVOS FL Auto Imaging microscope (Thermo Fisher) (Ita et al., 2020). Stained Safranin O/Fast Green articular surfaces (n = 3–6/rat) were scored by blinded graders using the modified Mankin score (Yeh et al., 2007; Ita et al., 2020). Regions of interest (ROIs; n = 2–4/image) throughout the images (n = 3-9 images/rat) were analyzed by Fourier transform (Sander and Barocas, 2009) to compute the principal orientation vectors of the image. The anisotropy index was calculated from the ratio of the principal axes to describe orientation on a continuous scale from isotropic (random; 0) to aligned (1) (Sander and Barocas, 2009) and averaged across ROIs for each rat to quantify collagen fiber orientation.

### Facet joint capsule biomechanical response to tensile loading

Harvested spines (occiput-T2) were dissected and the C6/C7 facet joints were isolated and finely dissected (Figure 1B) (Quinn and Winkelstein, 2007; Quinn et al., 2010). The *in situ* length across the C6/C7 motion segment was measured from the rostral-caudal midpoint of each vertebra’s laminae (Figure 1B and Table 1). Dissected spines were bisected, with one side prepared for biomechanical testing and the other side for the CHP assay; left and right sides were randomly assigned to each assay. For biomechanical testing, the capsular ligament of the isolated C6/C7 facet joint was marked with fiducial markers to enable strain tracking (Quinn and Winkelstein, 2007).

**TABLE 1:** Summary of *in situ* length, degraded collagen, macroscale biomechanics, maximum principal strain, microstructure, and anomalous events at mechanical events during tensile failure.

		Reference			First anomalous fiber realignment				Yield					
Rat		CV	CHP/mg	length	force	disp	MPS	CV	#Ev	force	disp	MPS	CV	#Ev
14	MMP-1	0.127	1191.7	2.25	0.177	0.771	0.082	0.141	1	1.255	1.648	0.165	0.165	2
15		0.063	450.0	2.82	0.478	1.664	0.126	0.115	1	2.589	2.622	0.254	0.147	5
19		0.083	747.4	3.34	0.490	0.889	0.104	0.139	1	0.910	2.355	0.154	0.147	3
55		0.119	796.3	2.66	0.065	0.738	0.105	0.157	1	1.161	1.783	0.210	0.148	4
56		0.149	1225.0	2.45	0.244	1.054	0.092	0.177	1	0.755	1.366	0.118	0.163	2
59		0.136	1437.2	2.27	0.546	1.069	0.117	0.216	1	2.521	2.918	0.380	0.195	4
	avg	0.113	974.6	2.63	0.333	1.031	0.104	0.158	1.00	1.532	2.115	0.213	0.161	3.33
	SD	0.033	369.5	0.41	0.198	0.339	0.016	0.035	0.00	0.813	0.608	0.094	0.019	1.21
54	vehicle	0.126	1710.0	3.13	0.132	0.637	0.110	0.188	1	1.341	1.587	0.224	0.202	3
57		0.102	560.7	2.66	2.208	0.750	0.138	0.122	2	2.791	1.088	0.197	0.189	8
58		0.098	963.6	2.88	1.449	0.844	0.071	0.159	1	2.006	1.374	0.121	0.188	2
61		0.093	574.2	2.33	0.462	1.881	0.194	0.143	1	0.539	1.934	0.287	0.138	2
62		0.156	786.3	2.74	0.823	0.551	0.188	0.179	2	1.441	0.789	0.269	0.205	1
	avg	0.115	918.9	2.75	1.015	0.933	0.140	0.158	1.40	1.624	1.354	0.220	0.184	3.20
	SD	0.026	472.4	0.29	0.827	0.542	0.052	0.027	0.54	0.836	0.442	0.066	0.027	2.77
65	naive	0.143	358.2	2.91	0.852	1.047	0.188	0.213	2	1.549	1.422	0.367	0.228	4

		First failure					Ultimate rupture					
Rat		force	disp	MPS	CV	#Ev	force	disp	MPS	CV	#Ev	stiffness
14	MMP-1	2.153	2.047	0.230	0.243	11	**2.153**	**2.047**	**0.230**	**0.230**	**11**	1.880
15		2.674	2.918	0.379	0.154	7	**2.674**	**2.918**	**0.379**	**0.154**	**7**	2.553
19		2.662	3.152	0.182	0.272	9	**2.662**	**3.152**	**0.182**	**0.272**	**9**	0.335
55		1.874	2.314	0.206	0.287	6	**1.874**	**2.314**	**0.206**	**0.287**	**6**	1.452
56		2.805	2.187	0.278	0.259	8	2.825	2.191	0.287	0.263	8	2.382
59		2.735	3.396	0.366	0.265	7	**2.735**	**3.396**	**0.366**	**0.265**	**7**	0.797
	avg	2.484	2.669	0.273	0.247	8.00	2.487	2.670	0.275	0.247	8.00	1.566
	SD	0.378	0.560	0.083	0.048	1.78	0.382	0.560	0.083	0.048	1.78	0.879
54	vehicle	2.348	2.406	0.263	0.266	6	2.451	2.770	0.609	0.281	11	1.740
57		2.791	1.088	0.197	0.189	8	2.966	1.165	0.312	0.206	9	4.424
58		2.958	2.951	0.251	0.254	8	**2.958**	**2.951**	**0.251**	**0.254**	**8**	1.255
61		2.044	2.587	0.225	0.263	9	2.301	2.859	0.273	0.282	7	1.519
62		1.536	0.835	0.289	0.248	6	1.730	1.158	0.305	0.255	8	2.584
	avg	2.335	1.973	0.245	0.244	7.40	2.481	2.181	0.350	0.256	8.60	2.304
	SD	0.574	0.949	0.035	0.032	1.34	0.515	0.933	0.147	0.031	1.51	1.285
65	naive	1.701	1.738	0.532	0.238	8	1.728	1.795	0.415	0.238	8	1.231

CV: circular variance; CHP: collagen hybridizing peptide; length is *in situ* reference in mm: force is in N; disp: displacement in mm; MPS: maximum principal strain; #Ev: number of anomalous events; stiffness is in N/mm; bold cells in ultimate rupture indicate capsules with first failure and ultimate rupture as a concurrent event.

The bold values in Table 1 indicate column headings.

Unilateral C6/C7 facets were mounted in an Instron 5865 (Instron; Norwood, MA) by gripping each of the laminae and transverse processes of the C6 and C7 vertebrae with micro-forceps (Figure 1B) (Quinn and Winkelstein, 2007). The *in situ* length was re-established in the loading device and taken as the unloaded reference position (Figure 1B). Tensile loading was imposed at 0.08 mm/s until visible tissue rupture. Force and displacement data were collected at 500 Hz along with high-speed imaging using a Phantom v9.1 camera (500 Hz; 40pixels/mm; Vision Research; Wayne, NJ). The integrated QPLI system acquired pixel-wise collagen fiber alignment maps before and during loading (Tower et al., 2002; Quinn et al., 2010; Zhang et al., 2016).

Force and displacement data were used to define events of interest during the failure test: yield, first failure, and ultimate rupture of the ligament (Figure 1B). The first failure was defined by the first decrease in force with increasing displacement before ultimate rupture (Figure 1B). Ultimate rupture was defined as the maximum force sustained during loading (Figure 1B). Yield was defined by the first occurrence of a decrease in the maximum tangent stiffness of at least 10% (Figure 1B) (Quinn et al., 2010). Ligament stiffness was calculated as a linear slope of the force-displacement curve fit from 20% to 80% of the force value at first failure (Figure 1B) (Ita and Winkelstein, 2019).

Collagen fiber alignment maps were generated during loading and used to determine the first occurrence of anomalous collagen fiber realignment (Quinn et al., 2010). Briefly, vector correlations were generated for every acquired alignment map to identify changes in alignment maps immediately preceding and following it based on pixel-by-pixel correlations (Quinn et al., 2010). Anomalous collagen realignment was defined by a decrease of 0.35 or more in the alignment vector correlation between maps, and a single region was defined as sustaining anomalous realignment when at least nine pixels were connected (Figure 1B) (Quinn et al., 2010). Alignment maps were also generated in the unloaded reference state to measure microstructural organization of each ligament prior to loading.

Force, displacement, collagen fiber alignment, and high-speed images were extracted at the first occurrence of anomalous fiber realignment, yield, first failure, and ultimate rupture. At the unloaded reference state and each of those events, fiducial marker locations were digitized. Marker coordinates were transformed into x-y coordinates using ProAnalyst (Xcitex, Inc.; Cambridge, MA) and maximum principal strain (MPS) was computed relative to the unloaded reference at each event in MATLAB (Matlab 7.2; Mathworks Inc., Natick, MA (Figure 1B) (Quinn and Winkelstein, 2007; Quinn et al., 2010). The number of anomalous events (Quinn et al., 2010) and the circular variance (CV) were calculated at each event, as well as at the reference state (Zhang et al., 2016; Ita and Winkelstein, 2019). CV quantified the spread of collagen fiber angles, with a lower CV indicating a higher degree of fiber alignment (Zhang et al., 2016; Ita and Winkelstein, 2019).

### CHP assay quantifying degraded collagen

To quantify the amount of degraded collagen, capsular ligaments from the C6/C7 facet joint were finely dissected and isolated. The wet weight of each isolated ligament was taken as the average of three measurements. Ligaments were lyophilized overnight and then incubated in 15 μM of 5-FAM conjugate of CHP (3Helix; Salt Lake City, UT) overnight, triple-washed in PBS for 30 min for each wash, then incubated in 1 mg/ml Proteinase K for 3 h at 60°C (Lin et al., 2019). After homogenization, the fluorescence of 200 μL duplicates of the homogenate solution was measured (Lin et al., 2019); fluorescence measurements were normalized to the wet weight of the sample as a metric of degraded collagen per ligament weight.

### Immunohistochemistry of substance P in neural tissue

To assess substance P expression in neural tissue, cryosections of C7 DRGs and spinal cord (n = 6/rat) were co-labeled with primary antibodies to microtubule associated protein (MAP-2) (chicken; 1:400; Abcam; Cambridge, MA) and substance P (guinea pig; 1:400; Neuromics; Edina, MN). MAP-2 was used to visualize neuronal somata and dendrites (Cullen et al., 2012). Immunolabeling was performed as described previously (Ita et al., 2020) with Alexa Fluor goat anti-chicken 488 and goat anti-guinea pig 633 secondary antibodies (1:1,000; Thermo Fisher). Tissue slides for which no primary antibodies were added were included to control for labeling procedures as controls to verify the specificity of each antibody.

Fluorescently labeled tissue sections were imaged with the 20x objective of a Leica TCS SP8 confocal microscope (n = 6-8 images/rat). The mean signal pixel intensity of substance P (n = 6-8 images/rat) was quantified in MAP-2-positive neurons (n = 10 neurons/image) identified by a blinded scorer (Ita et al., 2020). Neurons were categorized as small- (<21 μm), medium- (21–40 μm), or large- (>40 μm) diameter neurons (Weisshaar et al., 2010; Kras et al., 2014; Ita et al., 2020) to compare protein expression by neuron size. Spinal cord images were cropped to isolate the superficial dorsal horn (700 × 300 pixels); substance P was quantified by counting the number of pixels above the threshold for expression in naïve tissue using a custom MATLAB densitometry script (Weisshaar et al., 2010; Ita et al., 2020).

### Staistical analyses

Statistical analyses were performed with *α* = 0.05 using JMP Prov14 (SAS Institute Inc.; Cary, NC). Normality was tested using a Shapiro-Wilk test on the residuals of outcomes; non-normal outcomes were tested with non-parametric tests. A single rat was taken as the experimental unit. The average PWTs were compared between groups using a repeated-measures ANOVA with post-hoc Tukey HSD tests. Differences between Mankin score and anisotropy index were assessed with Wilcoxon Rank Sum tests. Separate repeated-measures ANOVA with post-hoc Tukey tests assessed differences in force, displacement, strain, CV, and the number of anomalous events across events within the injection groups, separately; comparisons for those same outcomes between groups were tested with Wilcoxon tests. Separate t-tests compared stiffness and CHP fluorescence between groups. Correlations between CHP and CV at sub-failure mechanical events were separately analyzed using linear regressions and an ANOVA for goodness-of-fit. Differences between groups for DRG substance P were tested using Wilcoxon tests. Separate t-tests assessed differences in spinal substance P expression between groups.

## Results

### Intra-articular MMP-1 induces immediate and long-lasting behavioral sensitivity

**FIGURE 2:**
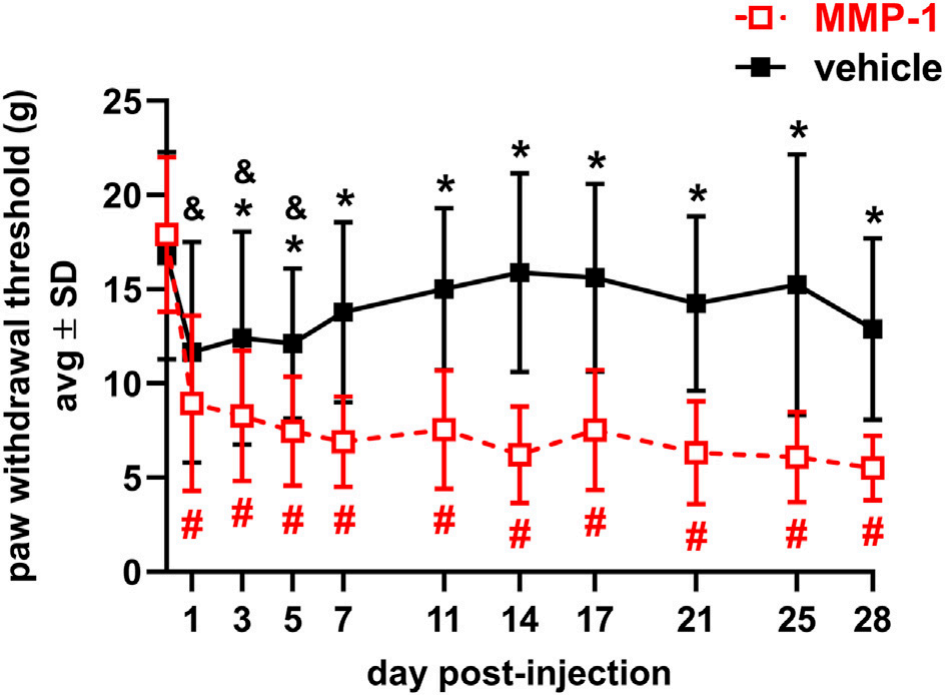
Paw withdrawal thresholds for 28 days after intra-articular injection of MMP-1 (n = 18) or vehicle (n = 13) with a decrease in threshold indicating greater sensitivity in the forepaw. MMP-1 decreases the threshold from baseline (day 0) for all days (#*p* < 0.0001) and decreases relative to vehicle responses beginning on day 3 (*p ≤ 0.0257). Rats initially show sensitivity after a vehicle injection, with decreased threshold relative to day 0 (&*p* ≤ 0.0273) but levels return to baseline in that group by day 7. Withdrawal thresholds at baseline (day 0) are not different between groups (*p* = 1.0000). Thresholds represent the average of both forepaws and all *p*-values are determined by repeated-measures ANOVA post-hoc Tukey tests.

MMP-1 decreases the PWT within 1 day of injection, lasting for at least 28 days (*p* < 0.0001) (Figure 2). That MMP-1-induced sensitivity is significantly different from the response to a vehicle injection beginning at day 3 and is sustained through day 28 (*p* ≤ 0.0257) (Figure 2). Although rats show sensitivity initially after vehicle injection (*p* ≤ 0.0273), it resolves by day 7. The PWTs at baseline before injection are not different between groups (*p* = 1.0000), making any differences after injection attributable to the injected agent itself (Figure 2).

### Joint tissue degradation is unchanged across the study groups, but is highly variable after MMP-1 injection

**FIGURE 3:**
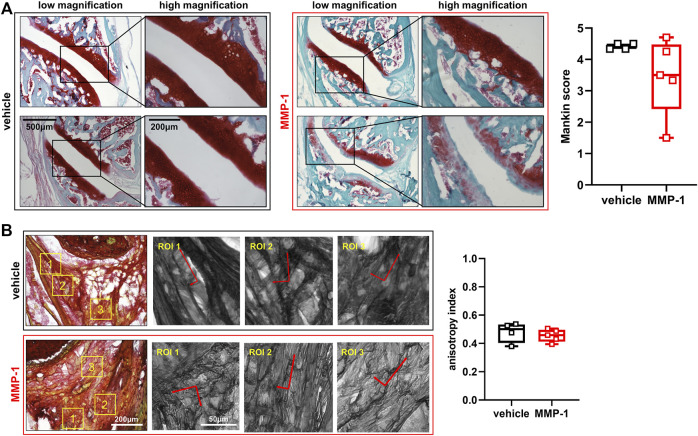
**(A)** Structural assessment of the facet joint cartilage and bone at 28 days after intra-articular injection (MMP-1 n = 6; vehicle n = 4). Low and high magnification images show overall healthy and non-degraded cartilage. The bottom panel of the MMP-1-injected joint shows lighter Safranin O staining and surface defects; that sample (Rat #37) has a 4.25 Mankin score. Degenerative features are not observed consistently across joint samples after MMP-1 injection; as such, there is no difference in Mankin score between groups (Wilcoxon Rank Sum test; *p* = 0.1057). A total of 3-6 surfaces were analyzed per rat **(B)** Evaluation of the collagen microstructure of the capsular ligament (MMP-1 n = 6; vehicle n = 4). Images (n = 3-9 images/rat) show Picrosirius Red-stained collagen fibers in the ligament. Yellow boxes show regions of interest (ROIs; n = 2–4/image) throughout each tissue section. The corresponding ROIs next to each stained image are overlaid with their corresponding principal orientation axes which were used calculate the anisotropy index, with one indicating aligned orientation and 0 indicating isotropic orientation. The anisotropy index is unchanged with MMP-1 (Wilcoxon Rank Sum test; *p* = 0.4489). The horizontal lines in the box-and-whisker plot represents the first (lower) quartile, median, and third (upper) quartile of the scores; whiskers represent the minimum and maximum of the data set. Individual data points are superimposed on boxplots. The scale bars on the low and high magnification images in **(A)** apply to all images with the corresponding magnification; the same applies for the images of stained ligament and their ROIs in **(B)**.

Despite significant changes in PWT, effects of intra-articular MMP-1 on the structure of joint tissues are not as evident. Joints injected with the vehicle appear healthy with normal Safranin O staining and no evidence of cartilage fibrillation (Figure 3A). Although there are occasional occurrences of lighter staining in the articular cartilage and mild surface fibrillations in joints injected with MMP-1 (Figure 3A), those observations are not consistent across the MMP-1 group. In fact, there is large variability in Mankin score with the MMP-1 injection, with no difference by injection agent (*p* = 0.1057) (Figure 3A). Like the Mankin score, the primary alignment of the collagen microstructure is unchanged in the ligament (*p* = 0.4489), with anisotropy indices the same between the MMP-1 (0.45 ± 0.18) and vehicle (0.47 ± 0.20) groups (Figure 3B).

### Ligaments undergo higher displacements in the sub-failure regime that suggest greater laxity after MMP-1

**FIGURE 4:**
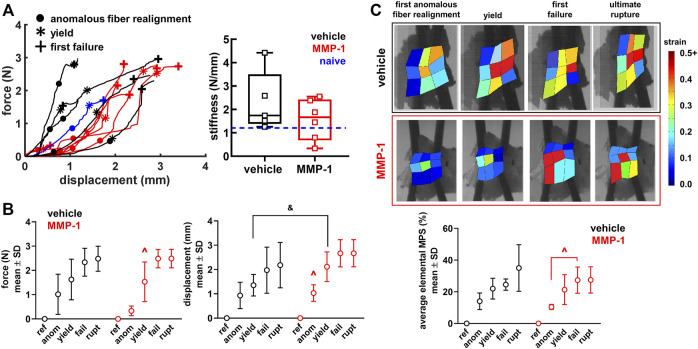
**(A)** Force-displacement responses showing the first occurrence of anomalous realignment (•), yield (*), and first failure (+). The curves end at ultimate rupture for each sample. Stiffness is not different between the MMP-1 (n = 6) and vehicle groups (n = 5) (*t*-test; *p* = 0.3127). The blue dashed line on the box-and-whisker stiffness plot shows the stiffness of a ligament from an un-operated rat **(B)** The force, displacement, and **(C)** average maximum principal strain (MPS) at each mechanical event show smooth progressions for the response of joints injected with vehicle and a step-like response for those with intra-articular MMP-1 (ref: reference; anom: first anomalous fiber realignment; yield; fail: first failure; rupt: ultimate rupture) **(B)** With intra-articular MMP-1, the force at yield (^*p* ≤ 0.0084) and the displacement at first anomalous realignment (^*p* ≤ 0.0070) are significantly different than at every other event (repeated-measures ANOVA with post-hoc Tukey HSD tests). Ligaments in the MMP-1 group have greater displacements at yield (Wilcoxon Rank Sum test; &*p* = 0.0404) than ligaments in the vehicle group **(C)** Exemplar full-field strain maps from a ligament injected with vehicle (Rat #62) and with MMP-1 (Rat #56) show that average MPS increases abruptly from first anomalous realignment to first failure with MMP-1 (repeated-measures ANOVA with post-hoc Tukey HSD tests; ^*p* = 0.0017), whereas the increase in MPS magnitude is more gradual with vehicle injection.

Ligaments exhibit a right-shifted biomechanical response after MMP-1 that suggests a weakened and altered biomechanical response (Figure 4A,B). The force-displacement responses of ligaments receiving a vehicle injection are like the response of the C6/C7 ligament from a naïve rat, which is within the range of the vehicle-injected capsules (Figure 4A). Ligaments receiving MMP-1, however, have curves shifted to the right, driven by a greater displacement at yield for the MMP-1-injected ligaments compared to the vehicle-injected ligaments (*p* = 0.0404) (Figure 4B and Table 1). Despite a nearly 1.5-fold decrease in stiffness with MMP-1 (1.56 ± 0.87N/mm), stiffness is not different from the vehicle group (2.30 ± 1.28N/mm; *p* = 0.3127) (Figure 4A and Table 1).

The altered biomechanical behavior induced by intra-articular MMP-1 is most pronounced in the sub-failure regime (Figure 4). Ligaments with a vehicle injection exhibit a smooth increase in force, displacement, and strain with the progression of the mechanical events (Figure 4). Intra-articular MMP-1, however, induces a “step-like” change at first anomalous fiber realignment and yield (Figure 4). For example, the force at yield with an MMP-1 injection is different from the force at every other mechanical event (*p* ≤ 0.0084); the same is true for displacements for intra-articular MMP-1 at the first occurrence of anomalous fiber realignment (*p* ≤ 0.0070) (Figure 4B). That step-like behavior is also evident for MPS (*p* = 0.0017) (Figure 4C). Such behavior is not observed for any outcomes (force, displacement, MPS) with intra-articular vehicle treatment (Figure 4).

### Microstructural ligament kinematics correlate with the extent of damaged collagen

**FIGURE 5:**
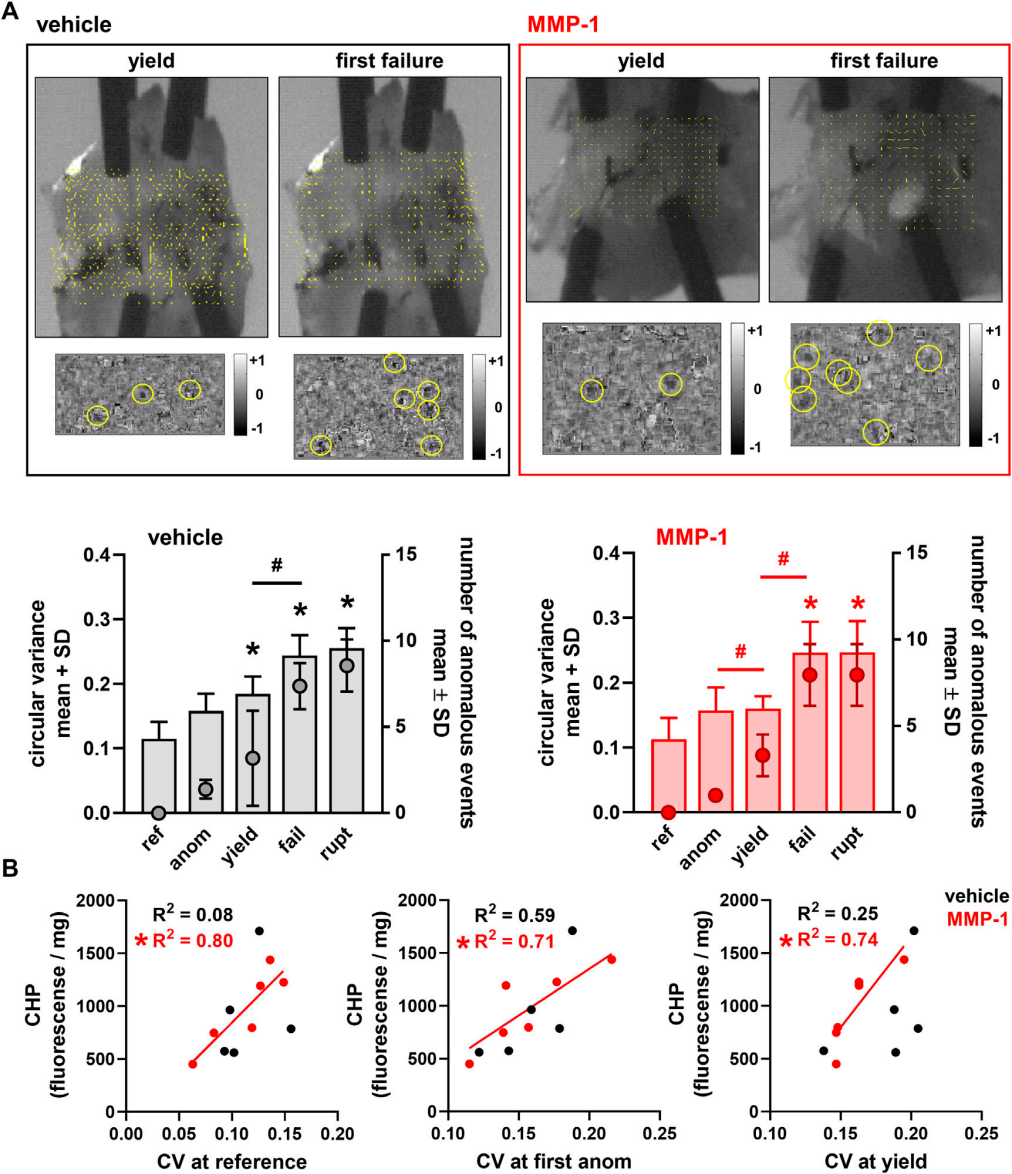
Collagen microstructural kinematics and relationships to the extent of degraded collagen at different mechanical events (ref: reference; anom: first anomalous fiber realignment; yield; fail: first failure; rupt: ultimate rupture) **(A)** Exemplar high-speed images with a corresponding collagen fiber alignment maps overlaid and the detection of anomalous fiber reorganization events (yellow circles in insets) at yield and first failure for ligaments injected with vehicle (Rat #54) or MMP-1 (Rat #56). Circular variance (bar plots; left axis) is different from reference at yield, failure, and ultimate rupture with the vehicle group (*p ≤ 0.0263 vs ref), and at failure and ultimate rupture relative to reference for the MMP-1 group (*p ≤ 0.0001). The number of anomalous events (mean ± standard deviation; right axis) is significantly different between yield and failure with vehicle (#*p* = 0.0031) and MMP-1 (#*p* = 0.0265) treatment; it is also different between the first occurrence of anomalous fiber realignment and yield in the MMP-1 group (#*p* < 0.0001). All *p*-values in **(A)** are calculated with separate repeated-measures ANOVA with post-hoc Tukey tests within the injection groups **(B)** The correlations between collagen hybridizing peptide (CHP) and circular variance (CV) show significant associations between CHP and CV with intra-articular MMP-1 at reference before loading (*p = 0.0157), at the first occurrence of anomalous fiber realignment (*p = 0.0350), and at yield (*p = 0.0279). However, those relationships are not significant for vehicle injection (reference: *p* = 0.6263; first anomalous realignment: *p* = 0.1256; yield: *p* = 0.3874). Correlations are separately analyzed using linear regressions; *R* ^2^ values show goodness-of-fit on each correlation plot.

There are subtle, but detectable, differences between groups in the microstructural kinematics across mechanical events. For intra-articular vehicle treatment, the CV at ligament yield differs from CV at reference (*p* ≤ 0.0263) (Figure 5A). However, for an MMP-1 injection, the collagen fibers do not reorganize until the ligament’s first failure (*p* ≤ 0.0001) (Figure 5A). Despite this, after an MMP-1 injection the number of anomalous events increases from the first occurrence of anomalous realignment to yield (*p* = 0.0263) (Figure 5A). In contrast, the number of anomalous events of vehicle-injected ligaments is not different between the first occurrence of anomalous realignment and yield, but differs between yield and first failure (*p* = 0.0031) (Figure 5A). The MMP-1-injected capsules also experience more anomalous events at failure than at yield (*p* < 0.0001) (Figure 5A).

In the reference configuration, neither the amount of degraded collagen measured by CHP fluorescence (MMP-1 974.6 ± 369.4; vehicle 918.9 ± 472.3; *p* = 0.8350) nor the microstructural organization of the capsule quantified by CV (MMP-1 0.112 ± 0.033; vehicle 0.115 ± 0.026; *p* = 1.0000) is different. Yet, the relationships between CHP and microstructural kinematics depend on whether the ligament was exposed to MMP-1 or vehicle (Figure 5B). There is a positive correlation between CHP and CV at reference for all ligaments (vehicle, MMP-1, and naïve) (*p* = 0.0482); yet, this correlation is driven by a significant association between CHP and reference CV that exists *only* for intra-articular MMP-1 (*p* = 0.0157; *R* ^2^ = 0.80) and not for intra-articular vehicle (*p* = 0.6263; *R* ^2^ = 0.08) (Figure 5B). That significant positive association between CHP and CV in the unloaded state with MMP-1 is maintained at both the first occurrence of anomalous fiber realignment (*p* = 0.0350; *R* ^2^ = 0.71) and yield (*p* = 0.0279; *R* ^2^ = 0.74) (Figure 5B).

### Intra-articular MMP-1 increases substance P expression in both the periphery and spinal cord

**FIGURE 6:**
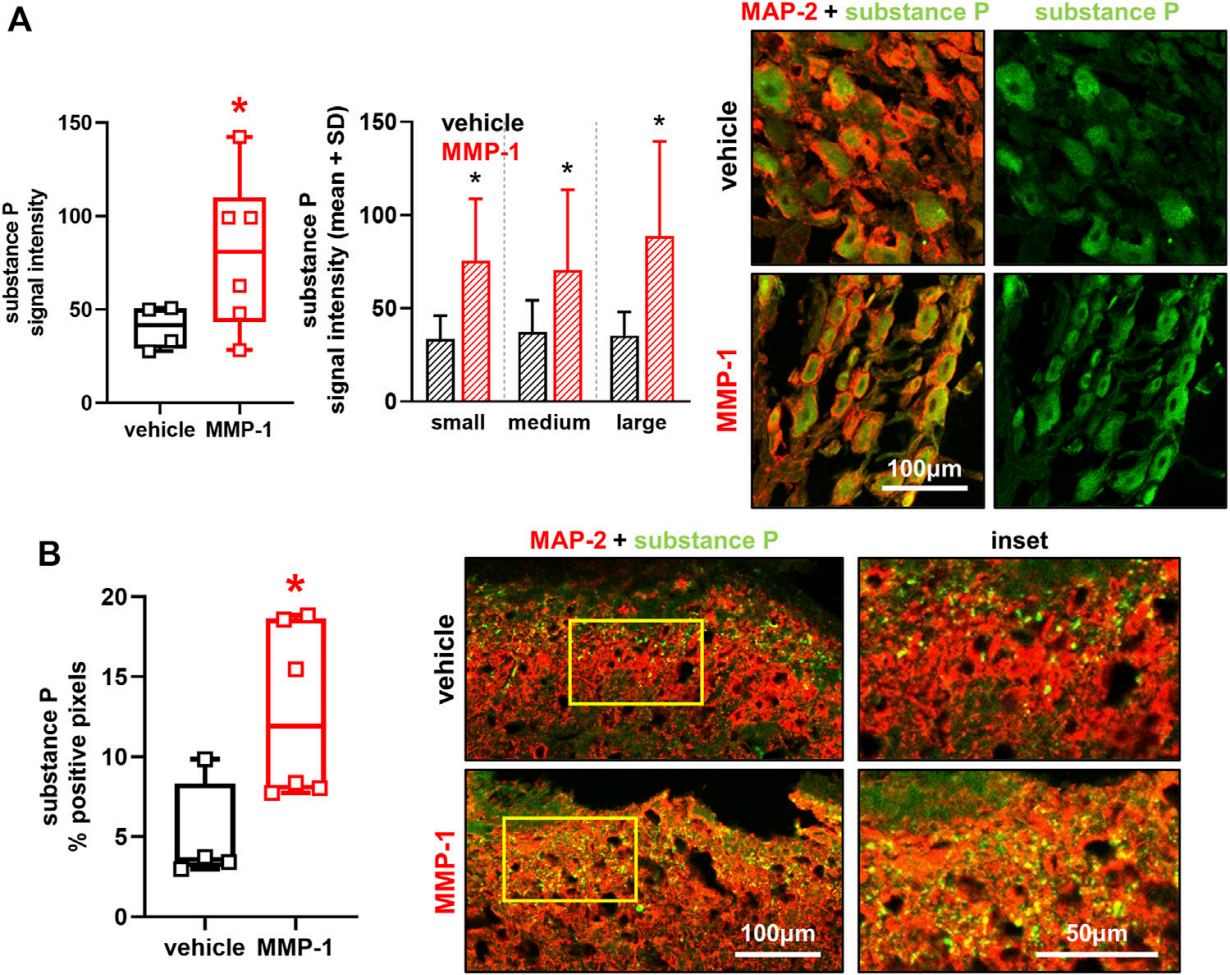
**(A)** Substance P labeling in dorsal root ganglia (DRG) neurons at day 28 after intra-articular vehicle (n = 4) or MMP-1 (n = 6). Separate images show immunolabeled DRG tissue sections with (left) channels for the neuronal marker MAP-2 (red) and substance P (green) merged (yellow) and (right) substance P only (green). Intra-articular MMP-1 significantly increases substance P (Wilcoxon Rank Sum test; *p < 0.0001) compared to vehicle treatment. The MMP-1-induced increase in substance P is evident in neurons of all sizes (Wilcoxon Rank Sum test; *p ≤ 0.0001). The scale bar applies to all images. Six to eight images (n = 10 neurons/image) were analyzed per rat **(B)** Labeling of substance P in the spinal superficial dorsal horn at day 28. Substance P labeling appears punctate and is increased with intra-articular MMP-1 (*t*-test; *p = 0.0020) over that with vehicle. The insets are magnified regions of the yellow boxes on the merged images. The scale bars also apply to the image directly above it. Box-and-whisker plot shows horizontal lines representing the first (lower) quartile, median, and third (upper) quartile of the data. Whiskers represent the minimum and maximum of the data set, with the mean value of images for each rat shown by individual data points that each represent the average of n = 6-8 images/rat.

Intra-articular MMP-1 significantly increases substance P expression localized to DRG neurons (*p* < 0.0001) in neurons of all sizes (*p* ≤ 0.0001) (Figure 6A). MMP-1 injection also increases substance P in the superficial dorsal horn at day 28 (*p* = 0.0020), with punctate labeling in most superficial layers of the dorsal horn (Figure 6B).

## Discussion

Intra-articular MMP-1 alone appears to be sufficient to induce immediate and sustained sensitivity in the rat (Figure 2), and the mechanism of MMP-1-induced sensitivity may involve altered capsular ligament microstructural kinematics and/or sensitization of DRG and/or spinal neurons (Figure 5, 6). The fact that sensitivity (Figure 2) and elevated substance P (Figure 6) occur in the absence of any significant modifications in the joint structure (Figure 3, 4) mirrors previous observations for intra-articular purified bacterial collagenase (Ita et al., 2020). These similarities suggest that exogenous proteases with collagenolytic functions have similar effects on tissue structure regardless of the protease source. Bacterial collagenase is hypothesized to induce sensitivity by degrading the collagen fibers in the ligament and subsequently triggering afferent signaling via altering the microenvironment of the afferents (Ita et al., 2020). This study provides additional biomechanical evidence supporting that hypothesis by demonstrating that MMP-1, likely via its collagenolytic capabilities, also alters collagen organization and reorganization during loading (Figure 5).

Given the large variability in Mankin score and instances of decreased Safranin O staining in cartilage after MMP-1 injection (Figure 2A), it is not possible to exclude the loss of cartilage ECM components as a possible mechanism of MMP-1-induced sensitivity. Safranin O staining is proportional to proteoglycan content in normal tissue and is a sensitive indicator of glycosaminoglycan (GAG) depletion (Schmitz et al., 2010). Indeed, proteoglycan loss has been shown to track with increasing pain-like behaviors after an injection of crude bacterial collagenase in the lumbar facet joints of rats (Yeh et al., 2008). Although the crude bacterial collagenase used in that study consists of a cocktail of proteases that cleave cartilage ECM components with much higher affinity than human MMP-1 (Fields, 2013), human MMP-1 does act on ECM substrates other than Type I collagen that are present in cartilage, such as Type II collagen (Visse and Nagase, 2003), and may thus affect cartilage composition directly. Alternatively, the altered biomechanics and microstructural kinematics observed after MMP-1 (Figures 5, 6) can alter force distribution at the joint’s articulation and cause downstream effects on cartilage health, a common progression in degenerative joint disease (O’Leary et al., 2018). Moreover, it is possible that variability in histological outcomes such as GAG depletion regionally track with the location of anomalous collagen fiber reorganization. Future experiments capable of pairing histology and collagen kinematics data may lend insight into this conjecture.

MMP-1 may degrade collagen in several regions of the capsule, predisposing ligaments to altered kinematics at both the macroscale and microscale (Figures 4, 5). It is possible that MMP-1 cleaves Type I collagen near the injection site and that those local regions respond differently to load than the surrounding unaffected neighboring regions. That notion is supported by CHP correlating with CV in the unloaded state after intra-articular MMP-1 (Figure 5B), since that positive correlation suggests that more damaged collagen fibers exist with more disorganized (less-aligned) fibers, even in an unloaded ligament. Since CHP measurements are made using whole capsular tissue homogenates, they lack the resolution to define regional variations. Nonetheless, this correlation holds at both the first detection of anomalous fiber realignment and yield (Figure 5B), supporting that the microstructural state of the facet capsule is different after MMP-1 exposure and remains that way during loading.

The proposal that the ligament is predisposed by MMP-1 degradation with “hot-spots” of anomalous collagen fiber reorganization may also explain the differential responses to loading between injected agents (Figures 4, 5). A greater displacement at yield for MMP-1 ligaments may indicate laxity (Figure 4) (Quinn and Winkelstein, 2011), which could explain the decreased PWT observed after MMP-1 (Figure 2) since normally physiologic movements could be painful. In fact, facet joint displacements that produce persistent pain symptoms also induce laxity and collagen fiber disorganization in the capsular ligament in the rat (Quinn et al., 2010). Furthermore, the occurrence of isolated, yet frequent, anomalous events with MMP-1 treatment (Figure 5) highlights the possibility that embedded afferents may be more susceptible to activation if they reside in regions where the collagenous matrix undergoes abnormal kinematics.

Since intra-articular bacterial collagenase may generate small collagen fragments via microscale collagen degradation of the collagen network (Ita et al., 2020) and this effect has been demonstrated with intra-articular MMP-3 (Otterness et al., 2000), it is possible that MMP-1 may generate small collagen fragments when injected into the joint space. Collagen fragments generated by MMP-1-mediated degradation can act as cell signaling agents on both fibroblasts and nerves in the ligament (Siebert et al., 2010; Leeming et al., 2011). For example, the collagen fragment known as C1M binds to integrin receptors that are expressed by fibroblasts and afferents (Siebert et al., 2010; Leeming et al., 2011). Integrin interactions at the cell surface can trigger intracellular signaling cascades, such as the activation of mitogen-activated protein kinase signaling pathways (Campos et al., 2004) and the dysregulation of neuropeptides (Zhang et al., 2017). The activation of those pathways by collagen degradation products may contribute to nociceptive transmission from intra-articular MMP-1 (Basbaum et al., 2009; Chen et al., 2015).

Load mediates the rate of enzymatic breakdown of collagen by protecting strained fibers from degradation (Ruberti and Hallab, 2005; Bhole et al., 2009). Since the collagen fiber network in the capsular ligament has varied orientations and undergoes heterogeneous strains under load (Ban et al., 2017), regions with collagen fibers that are *less* strained may be preferentially degraded by MMP-1. The preferential degradation of collagen fibers that are unstrained–bearing less load–could explain the simultaneous absence of overt structural damage with subtle, but measurable, effects of intra-articular MMP-1 on multiscale kinematics (Figures 3-5).

In addition to its regulation of the capsule’s collagenous network, MMP-1 could also induce nociception by initiating extracellular cell-signaling and/or intracellular protein regulatory pathways (Bartok and Firestein, 2010; Syx et al., 2018; Chakrabarti et al., 2020). For example, MMP-1 cleaves pro-inflammatory cytokines into their bioactive forms that can function as messengers in the extracellular space and cause abberent firing in peripheral neurons (Basbaum et al., 2009; Chakrabarti et al., 2020). There are positive feedback loops between MMPs and cytokines that are regulated by synovial fibroblasts and that lead to further production of MMPs, fibroblast infiltration, and inflammation (Bartok and Firestein, 2010; Sluzalska et al., 2017). Upon injection, MMP-1 may localize to fibroblasts and initiate the synthesis and activation of cytokines and/or other MMPs (Conant et al., 2004; Bartok and Firestein, 2010). This possibility is supported by the detection of cytokines in the synovial fluid of patients with painful osteoarthritis (Syx et al., 2018). Furthermore, inflammatory synovitis in the joints of patients with painful joint disorders correlates more strongly with reported pain than do structural changes like joint space narrowing and cartilage thinning (Emshoff et al., 2003; Torres et al., 2006; Hunter et al., 2013). So, although neither synovial tissue nor synovial fluid was assessed in this study, it is possible that the discordance between tissue-level evidence of degeneration and pain symptoms observed with intra-articular MMP-1 may be explained by inflammatory changes in the joint (Wang et al., 2018).

Both intra-articular MMP-1 and bacterial collagenase increase the expression of peripheral and spinal neuronal substance P (Figure 6) (Ita et al., 2020). Since both collagenases share collagenolytic activity, but do *not* share ECM-independent functional roles in cell signaling, the mechanism by which MMP-1 induces and mediates behavioral sensitivity likely is not completely independent of the ECM. In fact, it is possible that aberrant recruitment of mechanoreceptors may contribute to the sensitivity since substance P increases in large-diameter neurons (Figure 6). The recruitment of mechanoreceptors in pain processing only occurs in the presence of tissue injury (Obata and Noguchi, 2004). So, it is possible that the altered microstructural kinematics (Figure 5) injure proprioceptive fibers embedded in the collagen network. A loss of proprioception, and a corresponding *increase* in pain, is observed in patients with knee osteoarthritis (Malfait and Schnitzer, 2013), suggesting that mechanoreceptors may adopt nociceptive roles in states of degenerated pathology.

Increased peripheral and central substance P (Figure 6) suggests its role as a neuropeptide responsible for transducing nociceptive signals from the periphery to the spinal cord after MMP-1 injection; however, substance P is only one of the neurotransmitters released by peptidergic neurons in response to stimuli and/or neuronal injury (Pernow, 1953; Basbaum et al., 2009; Zieglgänsberger, 2019). Defining responses of other neuropeptides and neurotransmitters involved in pain, like calcitonin-gene related peptide (CGRP), galanin, glutamate, and adenosine triphosphate (ATP), would further inform the breadth of interaction of MMP-1 with the full pain axis. Indeed, studies inducing OA via monosodium iodoacetate injection in the rat report an increase in CGRP in primary neurons innervating the injected knee (Fernihough et al., 2005; Hong et al., 2020). Further, CGRP is upregulated in the capsular ligament of the hip joint in patients with painful osteoarthritis (Saxler et al., 2007). Together, these studies suggest that the neuropeptide CGRP also facilitates nociception from peripheral degenerated tissues.

Only male rodents were used in this study and as such, results do not capture any sex-based differences that may underlie the effect of MMP-1 on neuronal dysregulation and ligament kinematics. Nociceptive mechanisms depend on sex-based biological factors such as genetics and hormone levels, and also on experimental factors such as type of noxious stimulus, behavioral response being measured, and tissue type (Craft et al., 2004; Mogil, 2012; Sorge et al., 2015; Kumar et al., 2020; Liu et al., 2020). The progression of painful joint disease has also been shown to vary by sex in the temporomandibular joint (Fischer et al., 2008) and the knee (Bergman et al., 2021) in rodents. For example, post-traumatic knee osteoarthritis develops differently in male and female mice; both sexes develop pain-like behaviors with divergent synovial transcriptome profiles and males have a more severe histological phenotype (Bergman et al., 2021). Future experiments comparing outcomes between male and female rodents are necessary to fully elucidate the role of MMP-1 in joint pain.

Intra-articular MMP-1 alone may also induce sensitivity to thermal stimuli and/or mechanical stimuli beyond the sensitivity demonstrated using the von Frey filaments here (Figure 2). Although additional behavioral tests were not investigated in this study, time- and concentration-dependent effects of intra-articular bacterial collagenase have been observed using a range of pain-like behaviors. For example, intra-articular collagenase in the rat knee induces movement- and loading-induced nociception over a 6-weeks time course (Adães et al., 2014). In another study in rats, intra-articular collagenase in the knee significantly reduces paw-withdrawal latency to thermal stimuli for 8-weeks following injection (Lee et al., 2009). Additional behavioral assessments of the multi-modal manifestations of pain would help inform about the clinical condition of degenerative joint pain.

It is likely that the roles of exogenous human MMP-1 on Type I collagen and in non-ECM pathways in the rat facet joint mirror that of the MMP-1a rodent ortholog. In rodents, two MMP-1 orthologs to human MMP-1 have been identified, MMP-1a (Col-A) and MMP-1b (Col-B) (Balbín et al., 2001; Yurube et al., 2012). Due to its structural features, functional characteristics, and its demonstrated collagenolytic activity, MMP-1a has been hypothesized to represent the true ortholog of human MMP-1 (Balbín et al., 2001). Roles for MMP-1a in rodent models of pathology and disease states have further revealed analogous functions for MMP-1a in the rodent as are observed for MMP-1 in the human (Foley et al., 2013; Fletcher et al., 2021). Moreover, others have demonstrated that human MMP-1 activates rodent PAR-1 (Tressel et al., 2011; Foley et al., 2013), supporting the notion that the exogenous human MMP-1 in this study functions as the MMP-1a ortholog when injected intra-articularly. Studies including an experimental group with intra-articular injection of an inert *rodent* protein would help define whether findings are due to the mechanistic role of MMP-1 in the joint space or as a result of an immune response due to the use of a human protein. Nonetheless, the elevated pain response (Figure 2) and concurrent changes in ligament kinematics (Figure 5) and substance P expression (Figure 6) between MMP-1 and its paired vehicle group represent a first step in establishing MMP-1’s role in joint pain.

Collectively, findings support that increased MMP-1 in the joint space, over time, may predispose the collagen network to altered biomechanics that may alter the threshold for mechanically evoked pain. Indeed, the present findings align with the clinical presentation of degenerative joint pain whereby pain is experienced during normal activities and evidence of structural degeneration is subtle or absent (Hunter et al., 2013; Pan and Jones, 2018). As such, intervening in the MMP-1-mediated pathways may be particularly relevant for patients with that clinical presentation of joint pain.

## Data Availability

The raw data supporting the conclusions of this article will be made available by the authors, without undue reservation.
